# Oxyvinylenelactam Polymers—A New Class of Lactam-Based Kinetic Hydrate Inhibitor Polymers

**DOI:** 10.1021/acsomega.2c03644

**Published:** 2022-09-27

**Authors:** Malcolm A. Kelland, Radhakanta Ghosh, Audun Undheim, Erik G. Dirdal, Hiroharu Ajiro

**Affiliations:** † Department of Chemistry, Bioscience and Environmental Engineering, Faculty of Science and Technology, University of Stavanger, N-4036 Stavanger, Norway; ‡ Division of Material Science, Graduate School of Science and Technology, Nara Institute of Science and Technology, 8916-5 Takayama-cho, Ikoma, Nara 630-0192, Japan

## Abstract

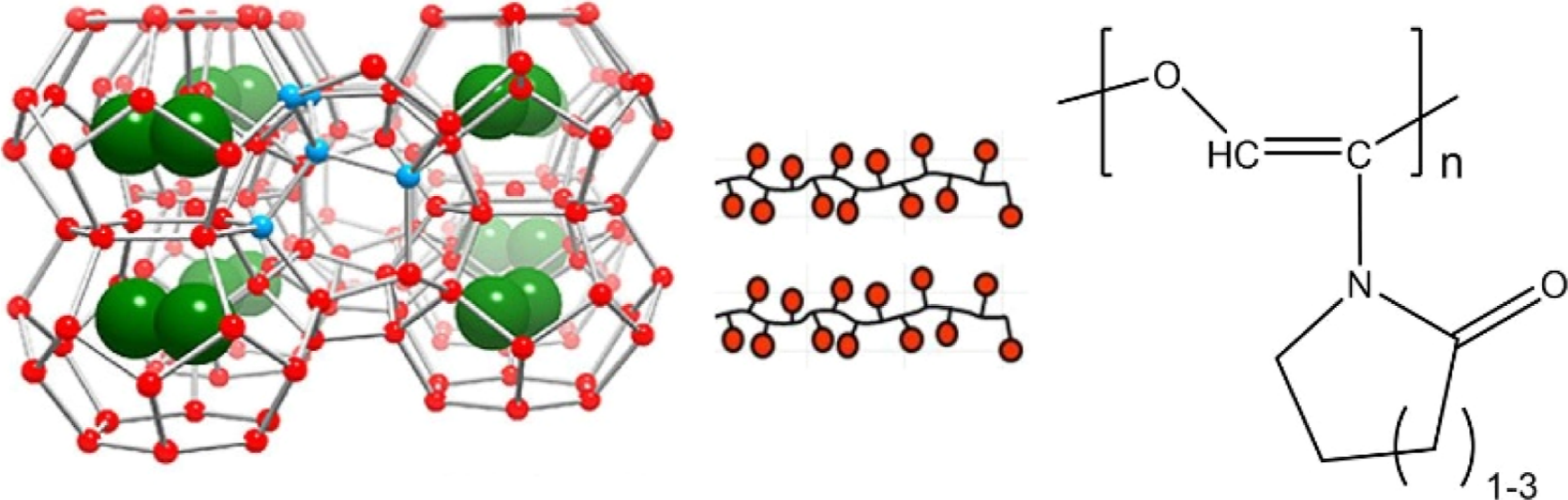

The deployment of kinetic hydrate inhibitors (KHIs) is a chemical method for the prevention of gas hydrate plugging in gas, condensate, and oil production flow lines. Polymers made using the monomer *N*-vinylcaprolactam (VCap) are one of the most common KHI classes. Alternative classes of polymers containing caprolactam groups are rare. Here, we present a study on oxyvinylenelactam polymers and copolymers with pendant piperidone or caprolactam groups. Low-molecular-weight homo- and copolymers were obtained. The nonrotating vinylene groups impart rigidity to the polymer backbone. Poly(oxyvinylenecaprolactam) (POVCap) was insoluble in water, but poly(oxyvinylenepiperidone) (POVPip) and OVPip:OVCap copolymers with 60+ mol % OVPip were soluble with low cloud points. KHI screening tests were carried out using the slow constant cooling method in steel rocking cells. POVPip was water soluble with no cloud point up to 95 °C but showed a poor KHI performance. In contrast, OVPip:OVCap copolymers with about 60–70 mol % OVPip were also water soluble and showed a reasonable KHI performance, better than that of poly(*N*-vinylpyrrolidone) but not as good as that of poly(*N*-vinylcaprolactam). Surprisingly, several additives known to be good synergists for VCap-based polymers showed negligible synergy or were antagonistic with the 62:38 OVPip:OVCap copolymer with regard to lowering the onset temperature of hydrate formation. However, a blend with hexabutylguanidinium chloride showed a strong effect to delay the onset of rapid hydrate formation.

## Introduction

**Figure 1:**
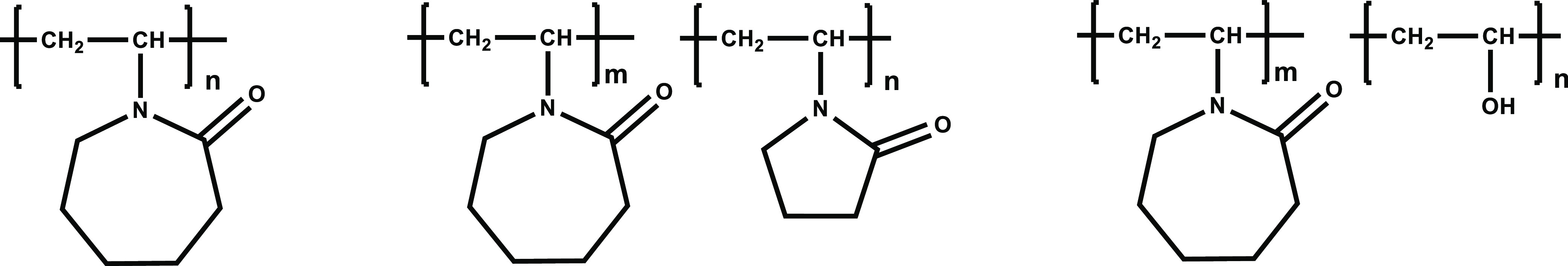
Examples of VCap-based KHI polymers. Left to right: PVCap, VCap:*N*-vinyl pyrrolidone copolymer (VCap:VP), and VCap:*N*-vinyl alcohol copolymer (VCap:VOH).

One of the most well-known kinetic hydrate inhibitors (KHIs) researched and used in the upstream oil and gas industry is poly(*N*-vinylcaprolactam) (PVCap) as well as VCap copolymers and graft polymers thereof (Figure 1).^1−15^

These polymers are usually used to prevent gas hydrate formation in unprocessed well stream fluids in flow lines, both subsea and on-land, under cold climate conditions. KHI polymers such as PVCap function by delaying hydrate particle growth whether as subcritical-sized particles (nucleation inhibition) or as crystal growth inhibition.^16^ The VCap monomer is affordable for the production of KHI polymers due to other larger applications such as personal care products.^17^

Despite their use in other applications, KHI polymers such as PVCap are still relatively expensive oilfield production chemicals.^18^ The VCap and VP monomers are made in very few places globally by the Reppe synthesis at a high temperature and pressure using ethyne.^19^ However, it would be useful to investigate the KHI performance of other classes of polymers containing caprolactam groups to find cheaper KHI polymers and ascertain if there is something unique about the PVCap structure that cannot be replicated. Therefore, alternate routes to PVCap or other polymers containing caprolactam ring structures have been sought.

**Figure 2:**
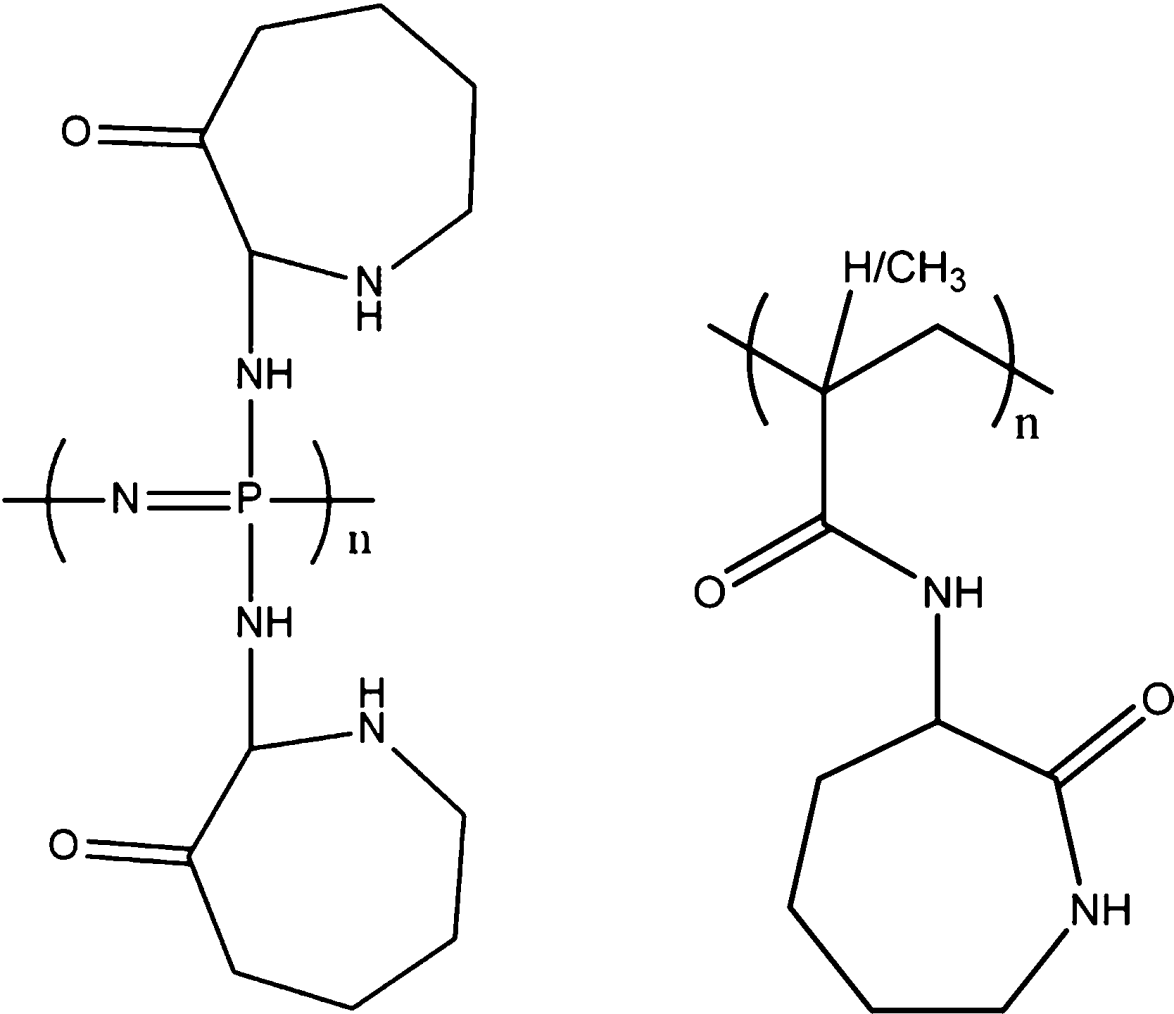
Structure of poly(caprolactam-2-amino)phosphazene (left) and poly(2-MACap (right).

Only a few studies on alternate polymers containing caprolactam rings as KHIs have been reported. 2-Aminocaprolactam has been the starting point for two classes of such polymers. The reaction with poly(dichlorophosphazene) gave poly(caprolactam-2-amino)phosphazene, which was water soluble as a homopolymer^20^ (Figure 2). This polymer showed some KHI activity effect but had some practical drawbacks. The reaction of caprolactam with polyamines and formaldehyde in a Mannich reaction was claimed to give useful KHI polymers with pendant caprolactam groups. The Mannich reaction is the amino alkylation of an acidic proton next to a carbonyl group, in this case, the caprolactam ring, by formaldehyde and the polyamine. However, we could not get this reaction to work, which was later unofficially confirmed by contacting the patent owners.^21^

The other class of polymer made using 2-aminocaprolactam is poly(2-methacrylamido-caprolactam) (poly-2-MACap) and the equivalent acrylamido polymers (poly-2-ACap).^22,23^ Both homopolymers were found to be insoluble in water, but a range of copolymers gave good performance as KHIs using a synthetic natural gas (SNG). Useful comonomers included *N*-methylmethacrylamide and *N*-vinyl-*N*-methylacetamide. The performance was also enhanced by synergists known to enhance the performance of PVCap, including isobutyl glycol ether (iBGE), 4-methyl-1-pentanol, tetrapentylammonium bromide (TPeAB), and hexabutylguanidinium chloride (Bu6GuanCl).

**Figure 3:**
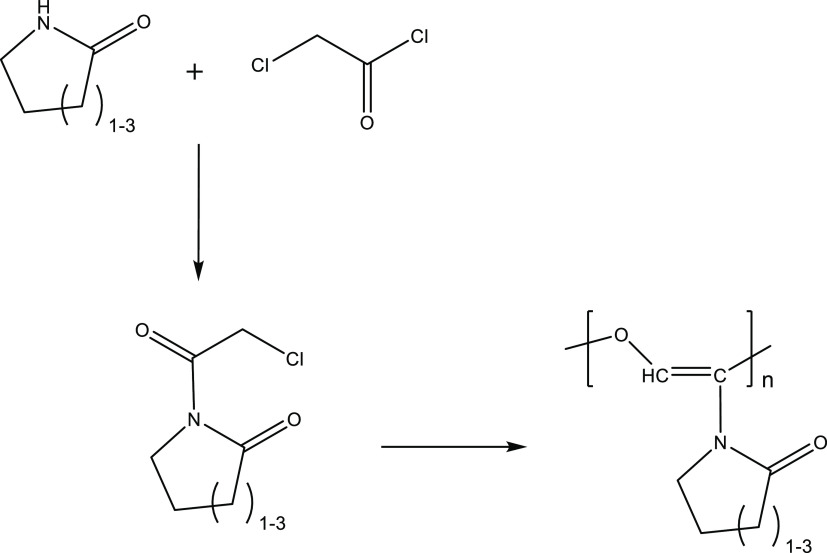
Synthesis of poly(oxyvinylene)lactams via 1-(2-chloroacetyl)lactams.

We have now investigated a new class of polymers with pendant caprolactam groups. These are poly(oxyvinylene)caprolactam copolymers made from the polymerization of *N*-(chloroacetyl)caprolactam and *N*-(chloroacetyl)piperidone (Figure 3).^24,25^ The poly(oxyvinylene)piperidone homopolymer was also investigated. KHI experiments were carried out in high-pressure rocking cells and compared to known *N*-vinyl lactam polymers.

## Experimental Section

### Materials

All chemicals were purchased from VWR (Avantor) and used as received. Poly(*N*-vinyl pyrrolidone) (PVP 15k, *M*_n_ 8000 g/mol) and PVCap homopolymers [*M*_n_ 2600 g/mol, 41.1 wt % in monoethylene glycol (MEG)] were kindly supplied by BASF. MEG in PVCap was removed before KHI testing by multiple precipitations from water above the cloud point (ca. 40 °C for a 1 wt % aqueous solution of the polymer).

### 1-(2-Chloroacetyl)lactam Monomer Synthesis

The synthesis has been illustrated for the caprolactam monomer. The synthesis of poly(1-oxy-3-lactam vinylenes) was based on the literature method.^24,25^ ε-Caprolactam and chloroacetyl chloride were mixed and stirred in a mole ratio of 2:1.126 in toluene under nitrogen in an ice bath. The solution was allowed to cool down slowly to room temperature over 1 h and left to react for 24 h. The solution containing caprolactam hydrochloride was filtered, and toluene was evaporated. This gave 1-(2-chloroacetyl)caprolactam with a yield of 85.0%. The same method was used to prepare 1-(2-chloroacetyl)pyrrolidone and 1-(2-chloroacetyl)piperidone. The ^1^H and ^13^C NMR data fitted the literature data.

### Synthesis of Poly(oxyvinylene)lactams

**Figure 4:**
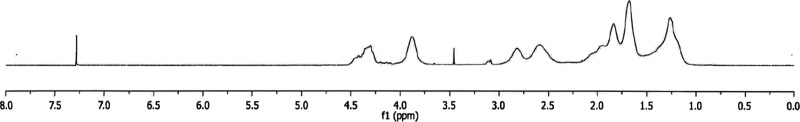
^1^H NMR spectra in CDCl_3_ of the POVCap homopolymer.

**Figure 5:**
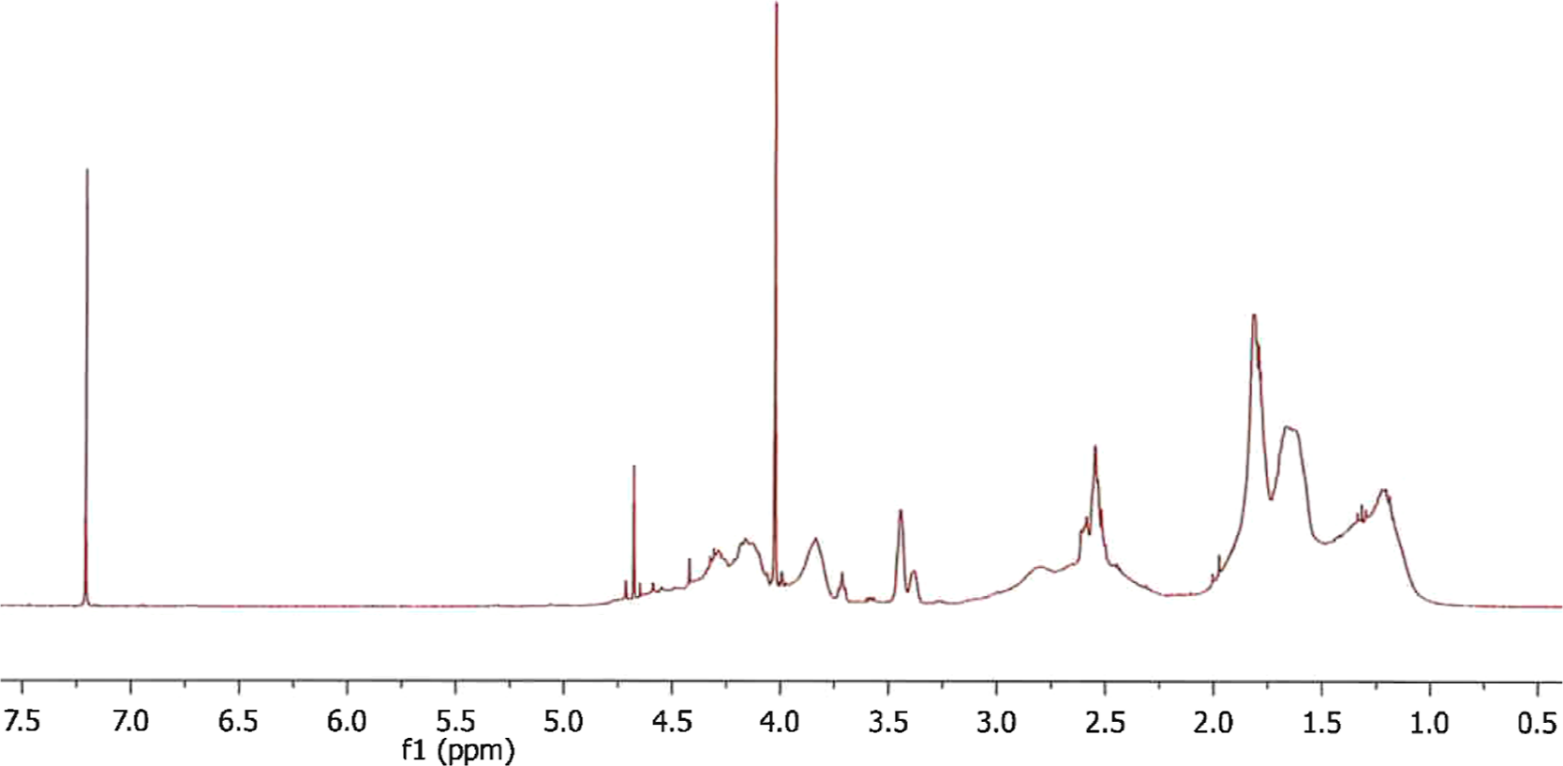
^1^H NMR spectra in CDCl_3_ of the 70:30 OVPip:OVCap copolymer. (The sharp peak at 4 ppm is an unidentified impurity.)

Polymerization of the monomers was done by adding a given amount of the monomer into a Schlenk flask which was then heated in an oil bath at 100 °C for 4 h under 20 mbar vacuum. ^1^H NMR spectroscopic analysis indicated 100% conversion. For the copolymers, this indicates that the starting monomer ratio is the same as the products. Figures 4 and 5 show the ^1^H NMR spectra in CDCl_3_ of the poly(oxyvinylenecaprolactam) (POVCap) homopolymer and 70:30 OVPip:OVCap copolymer, respectively. We are not sure what the sharp peak at 4.0 ppm is in Figure 5; it was not present in the 1-(2-chloroacetyl)caprolactam monomer spectrum. The resulting polymers were used directly in KHI experiments without further purification.

Gel permeation chromatography (GPC) molecular weight analysis was carried out using tetrahydrofuran (THF) as a solvent at 40 °C using superH3000 and GMH columns from Tosoh company and polymethyl methacrylate (PMMA) standards. The results are given in Table 1. The 80:20 OVPip:OVCap copolymer was not analyzed, but based on the other polymer results, it is also expected to have a *M*_n_ value of about 1000–1200 g/mol.

**Table 1:** GPC Results in THF with PMMA Standards^a^

polymer	*T*_Cl_ (°C)	*M*_n_ (g/mol)	PDI	comments
POVP				OVP does not polymerize
POVPip	<95	1000	1.09	
70:30 OVPip:OVCap	5–8	1100	1.10	
62:38 OVPip:OVCap	5	1200	1.12	deposition point 25 °C
50:50 OVPip:OVCap		1400	1.10	partially water soluble
POVCap 100%	<0	2700	1.06	insoluble in water

a PDI = polydispersity index.

### Cloud Point (*T*_Cl_) Measurement

The polymer was dissolved in deionized water to a concentration of 2500 ppm and heated slowly with shaking. The temperature at which clouding of the solution was first observed was taken as the cloud point (*T*_cl_). The test was repeated to check for reproducibility.

### KHI Performance Tests

**Figure 6:**
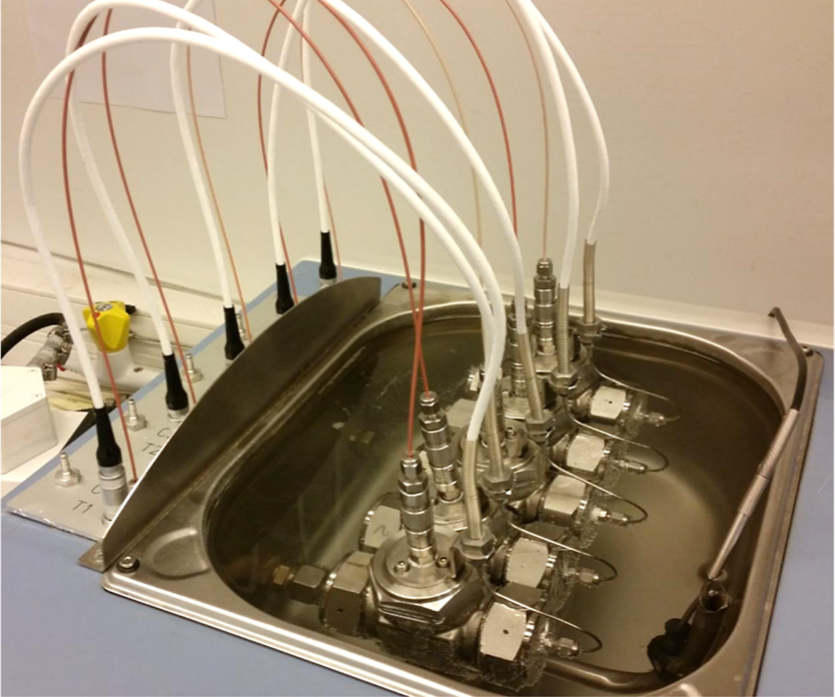
High-pressure steel multicell rocking in a temperature-controlled bath.

All KHI performance tests were performed in a series of five high-pressure 40 mL steel rocking cells. The cells were connected to a rocking axle placed in a thermally controlled water bath, part of the RC5 rig supplied by PSL Systemtechnik, Germany (Figure 6).^26^ Cells were pressurized with a SNG mixture, and the composition of the mixture is given in Table 2. This gas blend was made by Yara Praxair, Norway, and the composition was analyzed to be within ±0.1% of all the required concentrations. The hydrate equilibrium temperature (*T*_eq_) for sII gas hydrate at 76 bar of SNG was calculated to be 20.5 °C using PVTSim software (Calsep, Denmark).^27^

**Table 2:** Composition of the SNG Mixture

component	mol %
nitrogen	0.11
*n*-butane	0.72
isobutane	1.65
propane	5.00
CO_2_	1.82
ethane	10.3
methane	80.4

**Figure 7:**
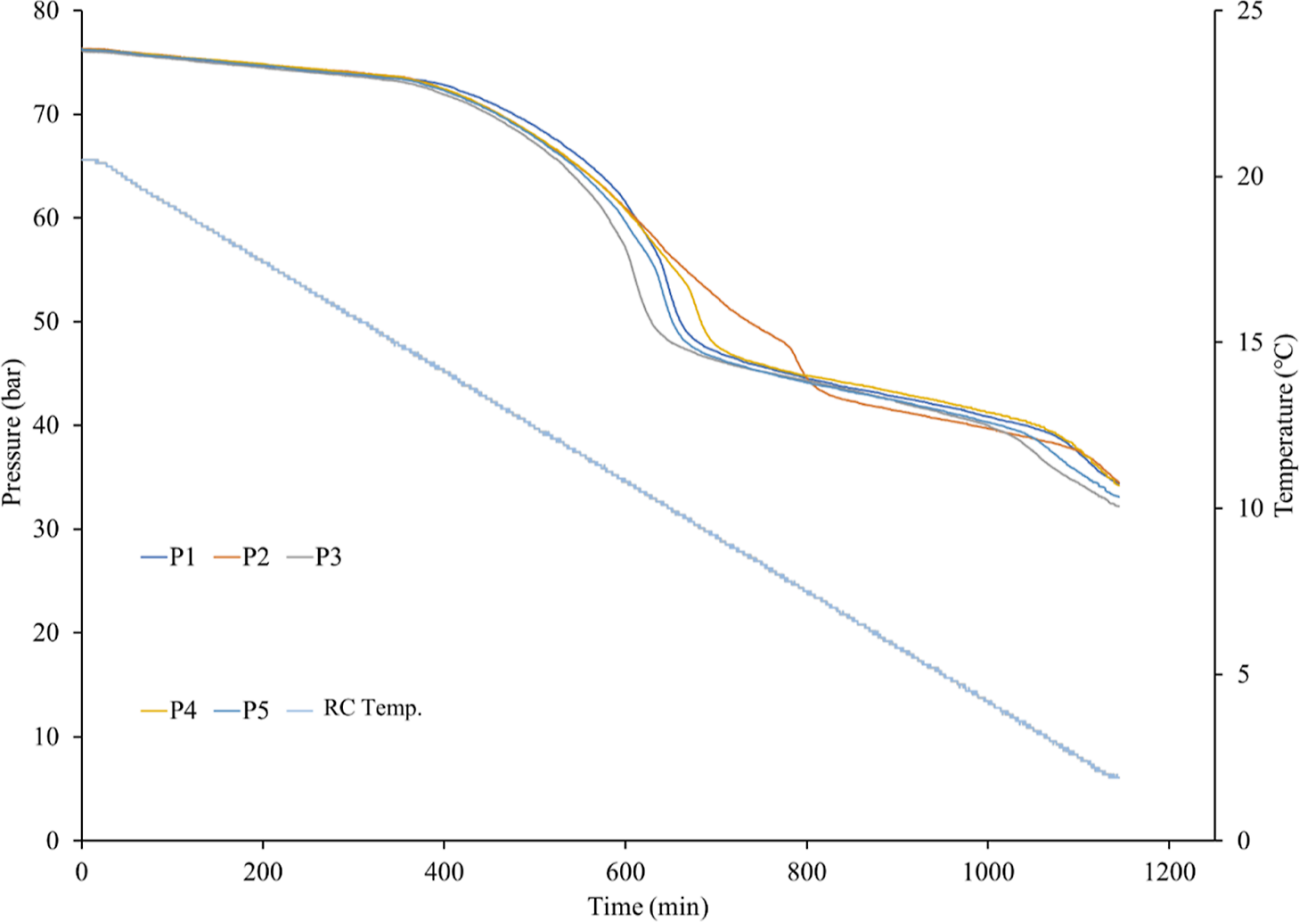
Example of pressure–time and temperature–time curves obtained from all five cells in SCC KHI screening tests (RC Temp. is the temperature in the cooling bath). This example is for a mixture of 2500 ppm OVPip:OVCap copolymer and 5000 ppm Bu6GuanCl.

Slow constant cooling (SCC) tests were carried out to evaluate the KHI performance of all polymers (Figure 7). This method has been used by our group for many years using the same equipment and SNG, which enables us to compare the performance of new KHIs to that of a plethora of previously tested KHIs.^28^ This was particularly useful for this study for comparison of the new polymer class with *N*-vinyl lactam polymers. The standard procedure for SCC tests was as follows:

1. The test polymer was dissolved in 105 mL of deionized water. Preparation was done 24 h prior to the KHI test. 20 mL of this test solution was added to each cell.
2. Each cell was purged with SNG, and then, vacuum was applied to remove air in the system. This was then repeated.
3. Approximately 76 bars of SNG was loaded into each cell at 20.5 °C, and each cell shut individually at the gas inlet/outlet valves.
4. The cells were rocked and slowly cooled at a rate of 1 °C/h. Pressure and temperature data were recorded using sensors.

**Figure 8:**
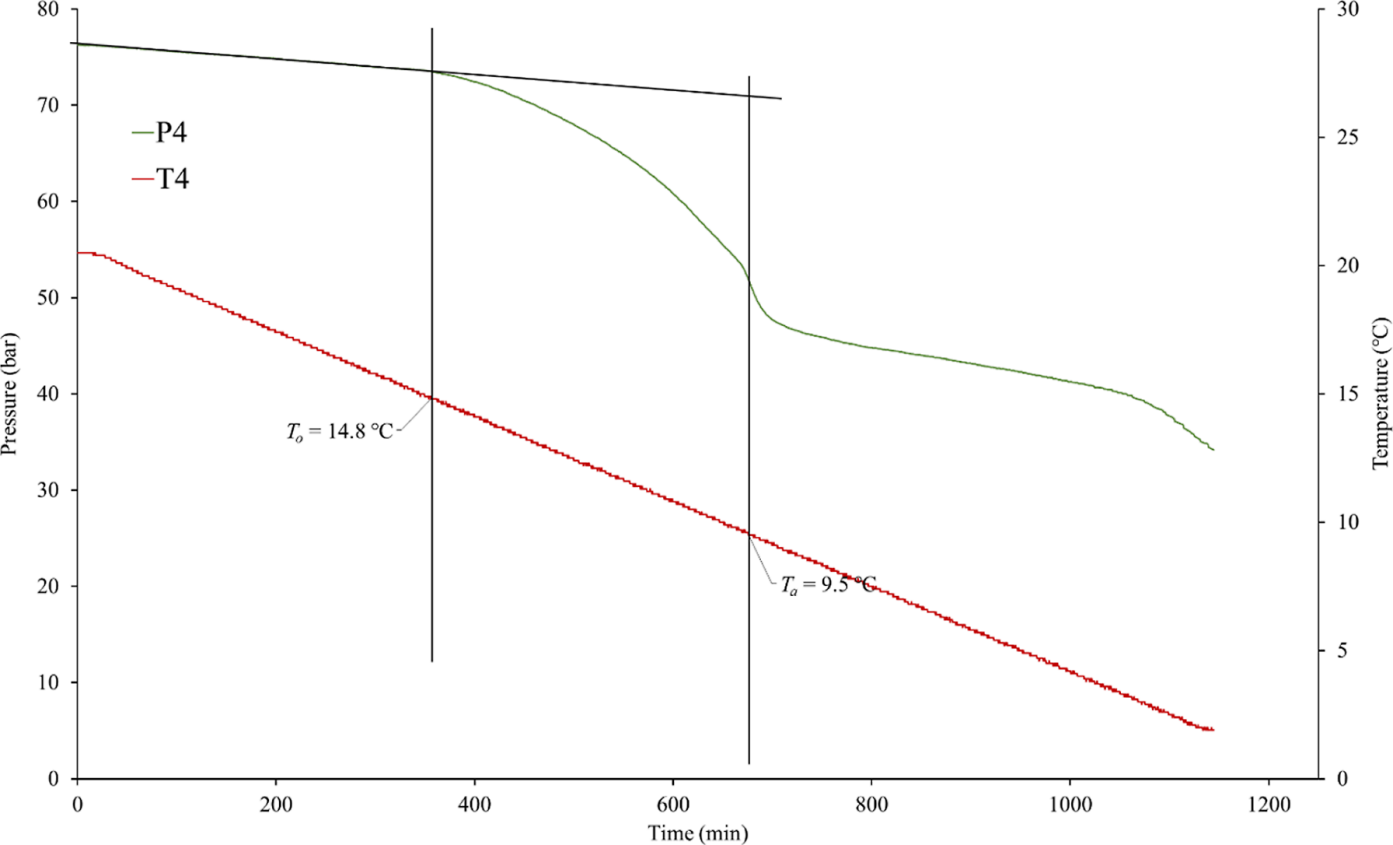
Determination of *T*_o_ and *T*_a_ values in cell 4 in a SCC KHI screening test.

An example of the data obtained (pressure and temperature vs time) from one experiment is shown in Figure 8.

From the SCC experiments, we derived two parameters: the hydrate onset temperature (*T*_o_) and the rapid hydrate formation temperature (*T*_a_) (Figure 8). As the system was closed, the pressure decreases linearly due to the constant temperature decrease. Once gas hydrates began to form, the pressure plot deviated from the linear track. At this point in time, the corresponding temperature was *T*_o_. The corresponding temperature at the start of the fastest pressure drop observed was marked as *T*_a_. Generally, 5–6 individual experiments were carried out for each polymer sample. For a set of 5–6 experiments, we typically observe 10–15% scattering in *T*_o_ and *T*_a_ values.^38^ This is due to the stochastic nature of the hydrate nucleation process. No bias was observed between any of the five cells, such as one cell regularly giving higher or lower *T*_o_ and *T*_a_ values than the other four.

## Results and Discussion

### Polymer Characterization and Water Solubility

Following the literature procedure, we were able to make all three lactam monomers, but in agreement with the original report, we could not polymerize the oxyvinylenepyrrolidone (OVP) monomer.^24,25^ Extending heating gave no sign of any change in viscosity or change in the ^1^H NMR spectrum. OVPip and OVCap were found to autopolymerize at room temperature but were stable when stored at 4 °C. All polymers made were orange-red colored. For those polymers that were water soluble, the solution at 2500 ppm was pale yellow-orange. The homopolymer POVCap was not water soluble. The ^1^H NMR showed complete conversion, so we assumed that the monomer ratios in the copolymers were the same as the initial ratios used. Also, in accordance with the literature, the poly(oxyvinylenepiperidone) (POVPip) homopolymer was only formed with a low molecular weight (*M*_n_ = 1000 g/mol). A similar level of polymerization was also seen for the OVPip:OVCap copolymers, whereas POVCap gave higher *M*_n_ values (2700 g/mol). This fits a trend of increasing polymerizability with increasing lactam ring size.

POVCap was not water soluble in the range of 500–5000 ppm and therefore was not tested as a KHI. The polymer might be dispersed in the aqueous phase from the turbulence in the flow line, but during shut-in, the polymer would sediment out and not be available to inhibit hydrate formation. POVPip was fully water soluble at 2500 ppm up to 95 °C. Therefore, we synthesized a range of OVPip:OVCap copolymers. POVPip forms a clear solution at 2500 ppm. The copolymers showed some opaqueness at this concentration, which gets stronger with increasing OVCap content until for the 1:1 copolymer, we observed some deposits. The rationale for these observations is as follows: assuming that the polymerization rates of the two monomers are different, there will be a distribution of comonomer ratios in any batch of the statistical copolymer. In addition, we know that the POVCap homopolymer is insoluble in water. Therefore, we assume the deposits formed from the copolymerization of OVPip with OVCap using 50% or more OVCap form some copolymer chains with too high a content of OVCap to give water solubility. In general, for the copolymers with 40 mol % or less OVCap, the cloud and deposition points increased with increasing mol % of the more hydrophilic OVPip monomer.

### KHI Performance

Table 3 gives a summary of SCC KHI performance screening results for the polymers in steel rocking cell tests using our SNG mixture. Tests with no additive and low-molecular-weight PVP and PVCap homopolymers were also included for comparison. In the table, we have also commented on the solubility and cloud and deposition points. The onset temperature *T*_o_ is considered the most valuable parameter as this represents the first detection of gas hydrate formation. The standard deviations are also given for this value. The *T*_o_*–T*_a_ value can also be useful to gauge the ability of the polymer to arrest hydrate growth. However, caution must be used in comparing data between polymers if the *T*_o_ values are considerably different since the driving force at the hydrate onset will not be the same.

**Table 3:** Average *T*_o_ and *T*_a_ Values for Five SCC Rocking Cell Tests with 2500 ppm Polymer Unless Otherwise Indicated^a^

entry	polymer	av. *T*_o_ (°C)	st. dev. for *T*_o_ (°C)	av. *T*_a_ (°C)
1	no additive	17.9	0.7	17.0
2	PVP (*M*_n_ 8000 g/mol)^29^	13.6	0.4	10.5
3	PVPip (*M*_n_ 3280 g/mol)^30^	10.5	0.3	9.2
4	PVCap (*M*_n_ 2600 g/mol)^27^	10.1	0.3	9.6
5	POVPip	13.9	0.4	13.7
6	OVPip:OVCap 80:20	13.9	0.3	13.8
7	OVPip:OVCap 70:30 (batch 1)	12.2	0.4	12.1
8	OVPip:OVCap 70:30 (batch 2)	12.1	0.3	12.0
9	OVPip:OVCap 62:38 (batch 1)	11.6	0.3	11.5
10	OVPip:OVCap 62:38 (batch 2)	13.0	0.6	12.8
11	OVPip:OVCap 50:50	13.3	0.3	13.3
12	POVCap-insoluble	not tested		

a Average of 10 tests for PVlactams referenced.

POVPip showed a weak KHI performance with an average *T*_o_ value of 13.9 °C over five tests. This is considerably worse than that of the low-molecular-weight poly(*N*-vinyl piperidone) homopolymer. We can only speculate a possible reason for this. The piperidone rings in POVPip are spaced further apart as there are three atoms in the polymer backbone compared to two for PVPip. This gives a lower density of active functional groups for POVPip. Second, the C=C double bonds in the backbone do not rotate, giving rigidity to the polymer. This may prevent the polymer from attaining a more optimal conformation for kinetic hydrate inhibition.

For the 62:38 OVPip:OVCap copolymer, we observed a discrepancy in the KHI performance between batches. The best batches gave average *T*_o_ values of 11.6 (five tests), whereas another batch gave a *T*_o_ of 13.0 °C. A possible reason is that the polymerization is done in bulk without a solvent and batches of different sizes. There may be poor mixing in the larger batch, giving a different range of copolymer ratios, although we could not observe any difference from the water solubility and *T*_Cl_ values. No discrepancy was seen for the 70:30 copolymer, which gave very similar KHI performances for both batches (an average *T*_o_ of 12.1 and 12.2 °C). In general, the 62:38 and 70:30 copolymers performed better than PVP but as well as PVPip or OPVCap. As for POVPip, the lower performance may be related to the density of lactam rings compared to that of poly(vinyl lactam)s (PVlactams) and the orientation of the backbone with nonrotating C=C double bonds.

Due to the low cloud and deposition points of the OVPip:OVCap copolymers, we attempted addition reactions with more hydrophilic molecules in the backbone vinyl group. This would hopefully enhance the water solubility as well as remove the lack of bond rotation with the vinyl C=C bond by creating C–C single bonds. This includes a reaction with hydrogen peroxide (catalyzed by transition metals), hydrogen sulfite addition, and the addition of iodine monochloride, followed by hydrolysis of the halide groups. However, so far, our attempts have failed to give a water-soluble polymer, probably because the oxyvinyl group is not stable under these conditions.

Vinyl lactam-based polymers have previously been shown to show a strong improvement in performance with increasing concentration. This might be related to their powerful ability to inhibit gas hydrate growth, compared to that of many other classes of amphiphilic KHI polymers including poly(*N*-isopropylmethacrylamide). Therefore, we were interested in determining the change in KHI performance with concentration for the vinylenelactam polymers. We tested a 62:38 OVCap:OVPip copolymer (*M*_n_ 1200 g/mol) at concentrations of 1000, 2500, and 5000 ppm and compared this to a low-molecular-weight PVCap. The results are summarized in Table 4. At equivalent concentrations, PVCap showed a better performance than the 62:38 OVCap:OVPip copolymer. Both polymers show a typical trend of increasing performance (decreasing *T*_o_) with increasing concentration. The differences between *T*_o_ and *T*_a_ values for the new copolymer are very small, indicating poor ability to arrest hydrate formation at the crystal growth stage.

**Table 4:** SCC Rocking Cell Test Results for 62:38 OVPip:OVCap at Varying Concentrations

	concentration ppm	av. *T*_o_ (°C)	st. dev. for *T*_o_ (°C)	av. *T*_a_ (°C)
no additive		17.2	0.7	16.6
PVCap	1000	12.9	0.3	12.0
	2500	10.1	0.3	9.6
	5000	7.3	0.2	6.4
62:38 OVPip:OVCap	1000	14.4	0.2	14.2
	2500	13.0	0.6	12.8
	5000	11.5	0.2	11.4

The performance of PVlactams is known to be enhanced by several classes of nonpolymeric molecules, including alcohols, glycol ethers, polyglycols, tetraalkylammonium salts, trialkylamine oxides, hexaalkylguanidinium halides, ionic liquids, and acetylenic diols.^31−41^ Therefore, we were interested in comparing the use of some of these synergists with the oxyvinylenelactam copolymers. The results are summarized in Table 5. We used the larger batch (ca. 2.0 g compared to 1.0 g for smaller batches) of the 62:38 OVPip:OVCap copolymer which gave an average *T*_o_ value of 13.0 °C with no synergists. Surprisingly, several additives known to be particularly good synergists for VCap-based polymers showed negligible synergy with the 62:38 OVPip:OVCap copolymer in extending the onset of hydrate formation to higher subcooling. Only the addition of tripentylamine oxide (TPeAO) showed a weak improvement in the KHI performance. However, Bu6GuanCl showed a strong ability to delay the onset of rapid hydrate formation as *T*_a_ dropped from 12.8 °C for the polymer only to 9.6 °C (Figures 7 and 8). Bu6GuanCl is known to be an excellent inhibitor of THF sII hydrate crystal growth.^37^ However, TPeAO and TPeAB also have the same effect, so we are unsure why only Bu6GuanCl showed this crystal growth KHI performance with the 62:38 OVPip:OVCap copolymer. We can only speculate as to why some additives showed poor synergy or even antagonism with the copolymer. For example, there may be some interaction between certain classes of synergists and the copolymer, as seen for some KHI polymers and corrosion inhibitors, which reduces the KHI performance.^42^ Molecular modeling might shed more light on this issue.

**Table 5:** KHI Test Results with 2500 ppm 62:38 OVPip:OVCap Copolymer with 5000 ppm Synergist

synergist	solution property	av. *T*_o_ (°C)	standard dev. (°C)	av. *T*_a_ (°C)
no synergist		13.0	0.5	12.8
iBGE	opaque	15.4	0.3	15.3
2,4,7,9-tetramethyl-5-decyne-4,7-diol	deposits	not tested		
TPeAB	opaque	13.7	0.2	12.4
Bu6GuanCl	opaque	14.8	0.3	9.6
4-methyl-1-pentanol (iHexOl)	opaque	16.3	0.3	12.7
TPeAO	opaque	12.3	0.4	11.2

## Conclusions

A series of low-molecular-weight poly(oxyvinylenelactam) homopolymers and copolymers were synthesized. POVCap was insoluble in water, but POVPip and OVPip:OVCap copolymers with 60+ mol % OVPip were soluble in water, giving opaque solutions and low cloud points. KHI screening tests using a sII-forming gas mixture were carried out using the SCC method in steel rocking cells. POVPip showed a relatively poor KHI performance, but OVPip:OVCap with about 60–70 mol % OVPip showed a reasonable performance, better than that of low-molecular-weight PVP but worse than that of PVCap. Several additives known to be good synergists for VCap-based polymers showed little effect or were antagonistic with the 62:38 OVPip:OVCap copolymer. However, a blend with Bu6GuanCl showed a strong ability to delay the onset of rapid hydrate formation. The oxyvinylenecaprolactam copolymers in this study represent the fourth class of caprolactam-containing polymers studied as KHIs, but VCap-based copolymers still represent the class with the best KHI performance. This study highlights further that it is not straightforward to make alternative classes of KHI polymers with caprolactam groups but that PVCap and its copolymers have a particularly useful structural motif for use as KHIs. Structural features that can be helpful to explain this are the high density of rings along the polymer chain and short distance of the caprolactam from the backbone.
